# Phage display of functional αβ single-chain T-cell receptor molecules specific for CD1b:Ac_2_SGL complexes from *Mycobacterium tuberculosis*-infected cells

**DOI:** 10.1186/1471-2172-14-S1-S2

**Published:** 2013-02-25

**Authors:** Frank Camacho, Jim Huggett, Louise Kim, Juan F Infante, Marco Lepore, Viviana Perez, María E Sarmiento, Graham Rook, Armando Acosta

**Affiliations:** 1 Finlay Institute, Havana, Cuba; 2 UCL, London, UK; 3 Experimental Immunology, University Hospital, Basel, Switzerland

## Abstract

The development of molecules specific for *M. tuberculosis*-infected cells has important implications, as these tools may facilitate understanding of the mechanisms regulating host pathogen interactions *in vivo*. In addition, development of new tools capable to targeting *M. tuberculosis*-infected cells may have potential applications to diagnosis, treatment, and prevention of tuberculosis (TB). Due to the lack of CD1b polymorphism, *M. tuberculosis* lipid-CD1b complexes could be considered as universal tuberculosis infection markers. The aim of the present study was to display on the PIII surface protein of m13 phage, a human αβ single-chain T-cell receptor molecule specific for CD1b:2-stearoyl-3-hydroxyphthioceranoyl-2´-sulfate-α-α´-D-trehalose (Ac_2_SGL) which is a complex presented by human cells infected with *M. tuberculosis*. The results showed the pIII fusion particle was successfully displayed on the phage surface. The study of the recognition of the recombinant phage in ELISA and immunohistochemistry showed the recognition of CD1b:Ac_2_SGL complexes and cells in human lung tissue from a tuberculosis patient respectively, suggesting the specific recognition of the lipid-CD1b complex.

## Background

TB, caused by *M. tuberculosis* (Mtb), remains a leading cause of morbidity and mortality, being responsible for more than 2 million deaths every year [1]. Prevention of transmission remains one of the most effective strategies and therefore new tools useful for early and rapid diagnosis are urgently needed. Assays currently used to detect specific T cell responses have shown several problems including lack of response to used antigens, variations due to concomitant infections and, last but not least, lack of evidence of current presence of the infectious agent [2]. Lipids, glycolipids and lipopeptides derived from Mtb are presented to T cells by non-polymorphic CD1 cell-surface molecules [3,4], thus expanding the possible targets available to the adaptive immune system for controlling the infection. Presentation of lipid antigens to T cells has been described in TB mediated by the presentation of lipids in association with CD1b molecules [5]. A novel lipid antigen belonging to the group of diacylated sulfoglycolipids purified from Mtb (Ac_2_SGL) has been identified. That Ac_2_SGL presented by CD1b molecules is recognized by specific T cells that are present in TB patients and PPD-positive, but not in PPD-negative individuals [3]. Taking into account that CD1b molecules are not polymorphic, Ac_2_SGL is expressed only by virulent mycobacteria, and that lipids are not subject to mutations induced by selective pressure of the host immune system, the use of CD1b:Ac_2_SGL complex as universal marker of tuberculosis infection should be evaluated. Phage display has been instrumental for the success of antibody (Ab) technology [6], but not fully expanded to TCRs [7]. The aim of the present study was, to display a functional αβ scTCR recognizing the CD1b:Ac2SGL complex from *M. tuberculosis*, on the PIII protein of m13 phage.

## Materials and methods

Total RNA from the human αβ T-cell clone T Z4B27 recognizing the CD1b-Ac2SGL complex [3] was isolated using the RNeasy kit from Qiagen according to the supplied protocol. Total cDNA preparation was performed using oligo(dT) primer and random primers together. The TCR V-genes were amplified using selected primers [8].

TCR V-genes were sequenced by GeneArt (Germany). A designed single chain TCR gene was synthesized by GeneArt and cloned into the phagemid vector pHEN-1[9] with standard techniques. The clone pHEN1-TCR was transformed into a F´ suppressor TG1 *E.coli* strain to produce phage displaying the αβ single-chain T-cell receptor.

Ac2SGL and the synthetic sulfoglycolipid analog SGL12 were used to load recombinant human CD1b molecules [3].

For the recognition test, 2x10^8^ purified phage displaying αβ scTCR particles per well were tested by ELISA in plates coated with human recombinant CD1b-Ac2SGL complex, CD1b-SGL12, CD1b unloaded or Ac2SGL alone [10].

Additionally the reactivity of these molecules by the phages expressing the scTCR was checked through immunohistochemistry using paraffin-embedded lung tissues from HIV+ TB patients.

## Results and discussion

The ability to display functional T-cell receptors on the surface of bacteriophages could be used to develop new strategies for isolating TCRs with unique specificity or to carry out mutagenesis studies on TCR molecules for analyzing their structure–function. The sequences of variable genes of the TCR of the human T-cell clone T Z4B27, specific for *M. tuberculosis* sulfoglycolipid Ac2SGL, displayed by the CD1b molecule were obtained. To design a αβ scTCR the variable genes were linked by a flexible hydrophilic peptide consisting in glycine and serine (Gly_4_Ser)_3_, which make it flexible and resistant to proteases [6]. The phagemid pHEN1 was selected to display the scTCR linked to PIII protein of m13 phage. After gene cloning, a colony PCR was performed in order to select recombinant clones and the selected clones were confirmed by automated sequencing.

**Figure 1:**
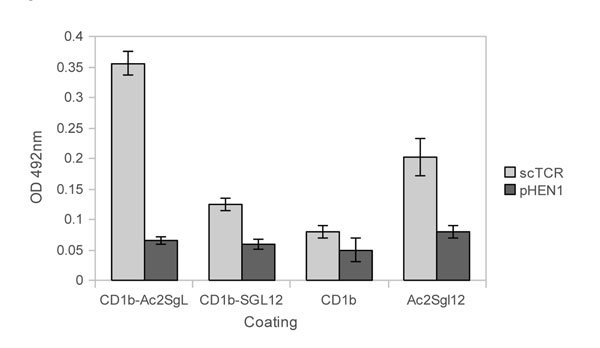
**Binding specificity of phage displaying αβ scTCR against CD1b-Ac2SGL complex.** Phages were detected using an anti-m13 antibody linked to Horseradish Peroxidase enzyme. The empty phage pHEN1 and unloaded CD1b were used as negative controls.

The phage displaying the αβ scTCR recognized the CD1b-loaded with Ac2SGL in ELISA (Fig.1). However, the recognition of CD1b loaded with the synthetic sulfoglycolipid analog SGL12 by the scTCR was lower than the one obtained with CD1b loaded with the natural sulfoglycolipid or Ac2SGL alone respectively (Fig.1). SGL12 is able to activate the T-cell clone T Z4B27 but with lower intensity than its natural counterpart Ac2SGL [11]. In this regard, it should be considered that the structures of Ac2SGL and SGL12 are different [11]. One of the differences is related with the -OH methyl groups of the lipid tails which plays an important role in the recognition of CD1b-lipid complex by the TCR[12].

T-cell activation needs the interaction of the TCR with the polar residues of lipids and with the amino acids from the CD1b-groove, so the recognition of free Ac2SGL by the scTCR (Fig.1) could be mediated by the interaction of the scTCR with polar structures of Ac2SLG recognized by the TCR of the T cell clone.

In general, the signals obtained in ELISA could be considered relatively low. These results could be explained if we take into account that the interaction of T cell receptors with their ligands are not of high affinity [13].

**Figure 2:**
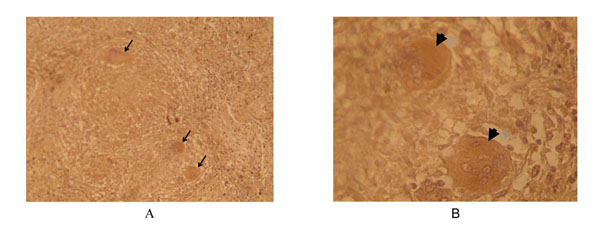
**Immunoperoxidase staining of a lung section from a TB patient by a phage expressing scTCR specific for CD1b-Ac2SGL complex. The phage displaying scTCR molecules were detected by staining with a mouse anti-m13 monoclonal antibody conjugated to horseradish peroxidase**. Positive cells are indicated by arrows. Micrographs are shown at a magnification of, A: 160 x ; B: 240 x.

The figure 2A shows multinucleated Langhans giant cells stained inside a defined granuloma formed by a central necrotic core surrounded by concentric layers of cells in a tissue section probed with the phage displaying the scTCR specific for the Ac2SGL:CD1b complex. No signal was detected using the control phagemid pHEN1. This result suggested the recognition of Mtb infected cells in tissues by the scTCR.

In summary our data show a functional TCR as a scTCR-PIII fusion displayed by the m13 phage that recognize the sulfoglycolipid antigen Ac2SGL from Mtb and discriminated between the CD1b loaded or unloaded with the Ac2SGL molecule. Additionally, the scTCR can differentiate the CD1b loaded with the natural antigen Ac2SGL or loaded with the synthetic analogue SGL12. The recombinant phage also recognized cells, probably infected with Mtb, in lung tissues from a HIV+ TB patient.

## Competing interests

The authors declare that they have no competing financial interests.

## Authors' contributions

All authors have read and approved the final manuscript. FC participated in the design and clonning of scTCR, in experimental design, immunological and histopathological studies, data analyses and in writing of the manuscript; JH participated in the design of clonning of scTCR; ML was involved in the production of CD1b-lipid complexes; JFI and VP performed the histopathological studies; GR participated in experimental design, data analysis and in writing of the manuscript; MES and AA conceived the study, participated in experimental design, histopathological studies, data analysis and in writing of the manuscript.

## Declarations

This article has been published as part of *BMC Immunology* Volume 14 Supplement 1, 2013: Proceedings from Delivery Systems and Current strategies to drug design. The full contents of the supplement are available online at http://www.biomedcentral.com/bmcimmunol/supplements/14/S1.
